# Patagonian sheepdog: Genomic analyses trace the footprints of extinct UK herding dogs to South America

**DOI:** 10.1371/journal.pgen.1010160

**Published:** 2022-04-28

**Authors:** Natasha Barrios, César González-Lagos, Dayna L. Dreger, Heidi G. Parker, Guillermo Nourdin-Galindo, Andrew N. Hogan, Marcelo A. Gómez, Elaine A. Ostrander

**Affiliations:** 1 Instituto de Farmacología y Morfofisiología, Facultad de Ciencias Veterinarias, Universidad Austral de Chile, Valdivia, Chile; 2 Escuela de Graduados, Facultad de Ciencias Veterinarias, Universidad Austral de Chile, Valdivia, Chile; 3 Departamento de Ciencias, Facultad de Artes Liberales, Universidad Adolfo Ibáñez, Santiago, Chile; 4 Center of Applied Ecology and Sustainability (CAPES), Santiago, Chile; 5 Cancer Genetics and Comparative Genomics Branch, National Human Genome Research Institute, National Institutes of Health, Bethesda, Maryland, United States of America; 6 Division of Biotechnology, MELISA Institute, Concepción, Chile; Queen Mary University of London, UNITED KINGDOM

## Abstract

Most modern dog breeds were developed within the last two hundred years, following strong and recent human selection based predominantly on aesthetics, with few modern breeds constructed solely to maximize their work potential. In many cases, these working breeds represent the last remnants of now lost populations. The Patagonian sheepdog (PGOD), a rare herding breed, is a remarkable example of such a population. Maintained as an isolated population for over 130 years, the PGOD offers a unique opportunity to understand the genetic relationship amongst modern herding breeds, determine key genomic structure of the founder PGOD populations, and investigate how canine genomic data can mirror human migration patterns. We thus analyzed the population structure of 159 PGOD, comparing them with 1514 dogs representing 175 established breeds. Using 150,069 SNPs from a high-density SNP genotyping array, we establish the genomic composition, ancestry, and genetic diversity of the population, complementing genomic data with the PGOD’s migratory history to South America. Our phylogenetic analysis reveals that PGODs are most closely related to modern herding breeds hailing from the United Kingdom. Admixture models illustrate a greater degree of diversity and genetic heterogeneity within the very small PGOD population than in Western European herding breeds, suggesting the PGOD predates the 200-year-old construction of most pure breeds known today. We thus propose that PGODs originated from the foundational herding dogs of the UK, prior to the Victorian explosion of breeds, and that they are the closest link to a now-extinct population of herding dogs from which modern herding breeds descended.

## Author summary

Studies of canine breed formation and ancestry are hypothesized to reflect history and migration of human populations. The Patagonian sheepdog (PGOD) or “Barbucho” is a working dog endemic to the Patagonian region of Chile and Argentina, having been imported to the region from the United Kingdom in the mid-1800’s. While recognized locally as a distinct, purposefully bred dog variety, the PGOD is not recognized by any formal dog breed registry. We hypothesize that the PGOD originated from working farm dogs prior to development of modern herding breeds, resembling the common ancestor of the modern herding dog breeds originating in the United Kingdom. Using whole genome SNP data, we analyzed the population structure and interbreed relationships between PGODs and 175 recognized breeds. Our data implicates the PGOD, which has remained distinct from and unaltered by other modern breeds, as the closest living representative of the common ancestor of modern UK herding breeds.

## Introduction

Modern dog breeds result from human selection for traits reflecting both aesthetic values and the behavioral needs of human populations [1]. While most modern breeds were developed in Western Europe during the Victorian age by fanciers [2], many working breeds were developed using a deliberate two-step process; initial selection for functional traits designed to accomplish specific tasks critical to human survival, such as herding, hunting, and protection, followed by more recent prioritization of nuanced morphological attributes [3–5]. Restrictive geography and the specific needs of particular human enterprises, such as the livestock or sheep industry, have heavily influenced genetic variation within and among herding dog populations [6]. Livestock dogs, with herding and guarding aptitudes, are a particularly interesting example as they are required to fill multiple roles: protection, guarding, and guiding agricultural populations [7].

**Fig 1:**
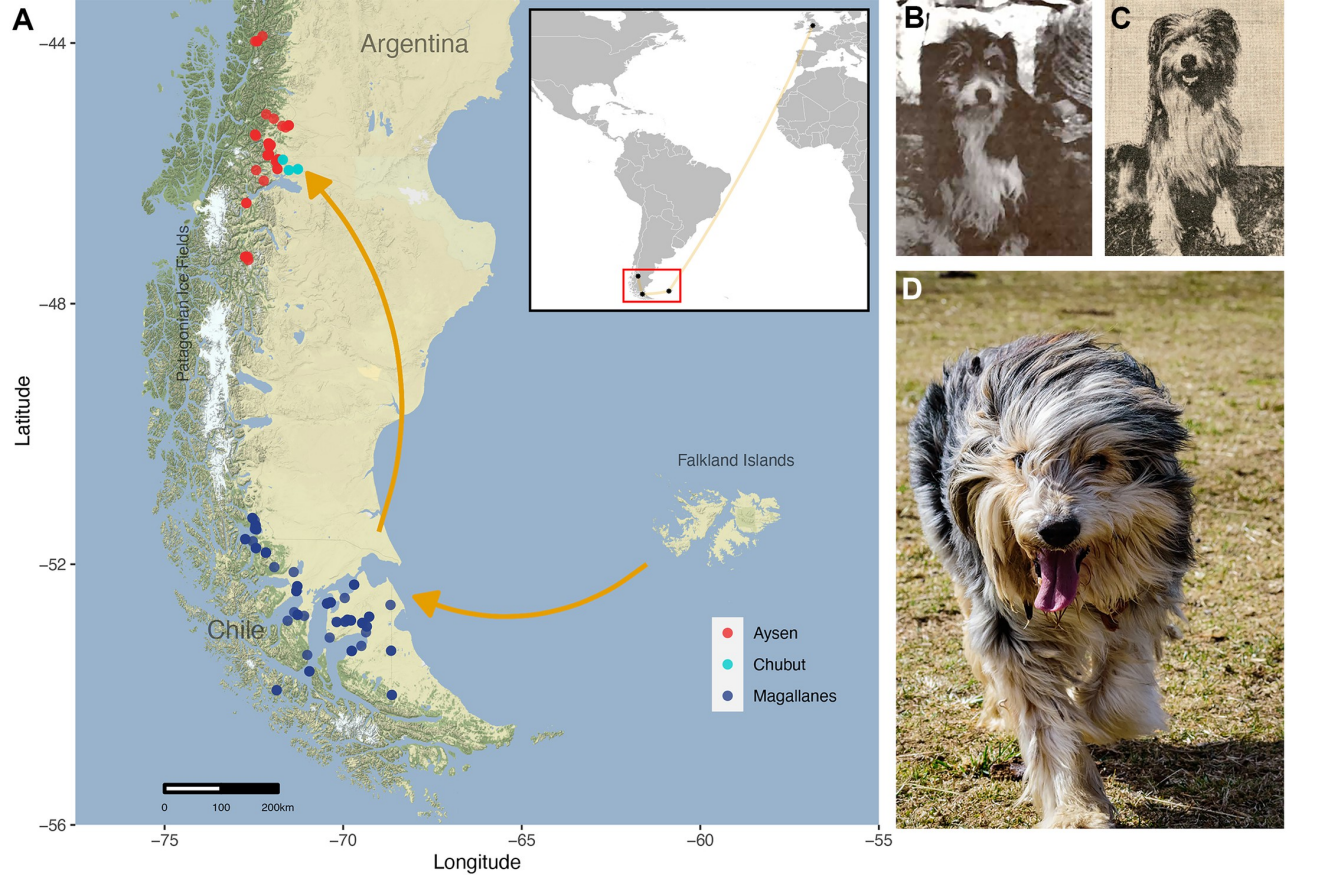
Historical migration route of Patagonian sheepdog. (A) The inset box shows the historical migration route (orange line) from UK to Falkland Islands in the South Atlantic Ocean, east of Argentina. Orange arrows in the main map indicate the subsequent migration of PGOD ancestors from Falkland Islands to southern and northern Patagonia. The Patagonian ice fields are labeled and visible as a white color on the main map. Each dot indicates the sampling locations of PGOD individuals used in this study (light blue: Chubut, Argentina; red: Aysén (Northern Patagonia) and blue: Magallanes (Southern Patagonia), Chile) (Map tiles by Stamen Design, under CC BY 3.0. Data by OpenStreetMap, under ODbL; http://maps.stamen.com/terrain/beta/#6/-52.407/-71.167). (B) An Old Welsh Grey dog, a type of herding dog indigenous to regions of Great Britain which is now extinct (by Carpenter B; http://www.bordercolliemuseum.org/BCCousins/ExtinctBreeds/ExtinctBreeds.html) (C) Type of dog that arrived to Patagonia during late 1800. The photograph is from the Magallanes region in Chile and was taken in 1933 [16]. (D) Photograph of a Patagonian sheepdog from the Aysén region of Chile, taken in 2020.

We and others have hypothesized that migration and ancestry of canines mirrors the history of human populations and their movements [6,8]. The Patagonian sheepdog (PGOD), also called the “Barbucho” or “Ovejero Magallánico”, is a working dog found in the Patagonian region of Chile and Argentina [9]. While recognized locally as a distinct, purpose-bred dog variety, the PGOD is not recognized by any formal dog breed registry, as is often the case with landrace breeds worldwide [6,10]. Historic documents indicate that the PGOD descended from working dogs brought by Scottish settlers who immigrated to Chilean Patagonia to develop sheep farming in the region, likely between 1877 and 1910 (Fig 1A) [9,11–13]. As official standards for individual collie-type breeds were not yet defined, dogs used in sheep farming in Great Britain at this time were simply known as “working collies” or “shepherd dogs” that had been adapted to terrain and climate (Fig 1B) [9,14]. The geographical isolation of Patagonia, strong behavioral selection over the past 130 years, and little to no introduction of new breeding stock has resulted in a distinct population, uniquely adapted to a harsh environment and the needs of the Patagonian people [9,15,16] (Fig 1C and 1D).

In this study we performed phylogenetic and haplotype sharing analyses to investigate the relationship between the PGOD and modern herding breeds originating in the United Kingdom (UK). We explored genomic structure and ancestry to determine the relationship of the PGOD with modern dog breeds. Using a measure of shared haplotypes, we estimated the divergence time of the PGOD relative to other UK herding breeds to produce a timeline of herding breed development. Our data suggest that the PGOD is likely the closest living representative of the common population from which modern UK herding breeds originated.

## Results

### Phylogenetic relationship among dog breeds

**Fig 2:**
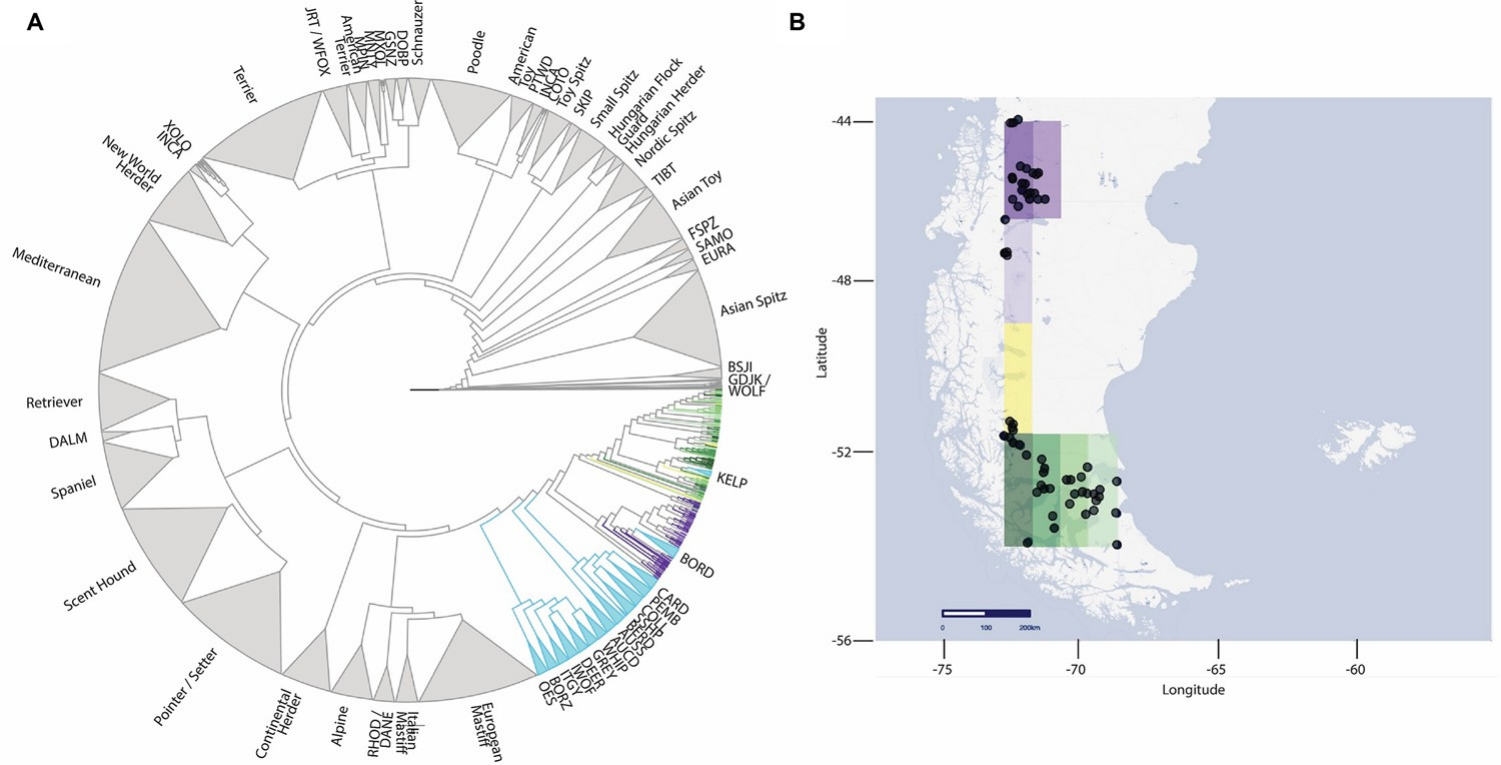
Phylogeny cladogram calculated by genetic distance. (A) Neighbor-joining cladogram of genetic distances, based on 100 bootstraps, using published data from 176 breeds, including PGODs, and two wild canids [5]. In the cladogram, individual dogs are clustered by breed if their bootstrap value is 100%, and breeds are condensed into clades, as defined previously [5], with breed-to-breed bootstraps of >90%. Light blue indicates UK rural breeds. Individual PGODs are shown as branches in shades of purple through green indicating the sampling locations. (B) Locations from which PGODs were sampled are indicated with black circles. The geographical area is divided into segments of equal size, with samples assigned to one of eight regions (Map tiles by Stamen Design, under CC BY 3.0. Data by OpenStreetMap, under ODbL; http://maps.stamen.com/toner-lite/#6/-49.803/-71.213). Dogs collected in each segment of B are represented using the same color coding as in A. List of the breeds in Fig 2A with their abbreviations can be found in S1 Table.

To identify the relationship between PGODs and other recognized breeds, we generated a cladogram with SNPs from 1673 individuals representing 176 dog breeds, including the PGOD, and two wild canids [5]. The cladogram was created using an identity by state (IBS) distance matrix and Neighbor Joining (NJ) phylogeny [17]. Using a consensus tree built with 100 bootstrap replicates, the PGOD clustered 92% of the time with a group of breeds termed the “UK rural clade”, one of the 23 phylogenetic clades previously identified [5]. This clade includes herding breeds with origins in the United Kingdom, e.g., Border collie (BORD), Shetland sheepdog (SSHP), old English sheepdog (OES), Pembroke welsh corgi (PEMB), and Australian shepherd (AUSS). Within the 16 member UK rural clade, PGODs are monophyletic with the BORD and the Australian kelpie (KELP) (Fig 2A). In addition, we observed a geographic pattern in the phylogeny such that most PGODs from northern Patagonia (the Aysén region in Chile, including seven dogs from the cross-border region of Chubut, Argentina) are more closely related to the BORD (100% bootstrap value), while PGODs collected in southern Patagonia (Magallanes region in Chile) are more closely related to the KELP (100% bootstrap value) (Fig 2B).

Interestingly, dogs from these two regions are not only geographically isolated from the rest of Chile (i.e., limited by channels and fjords to the north, the Pacific Ocean to the west, Argentina to the east and the Drake passage to the south) but are also separated by the north and south Patagonian Ice Fields (Fig 1A). Yet, despite their geographical separation, both PGOD populations are similar in terms of morphology and behavior.

### Shared haplotypes

**Fig 3:**
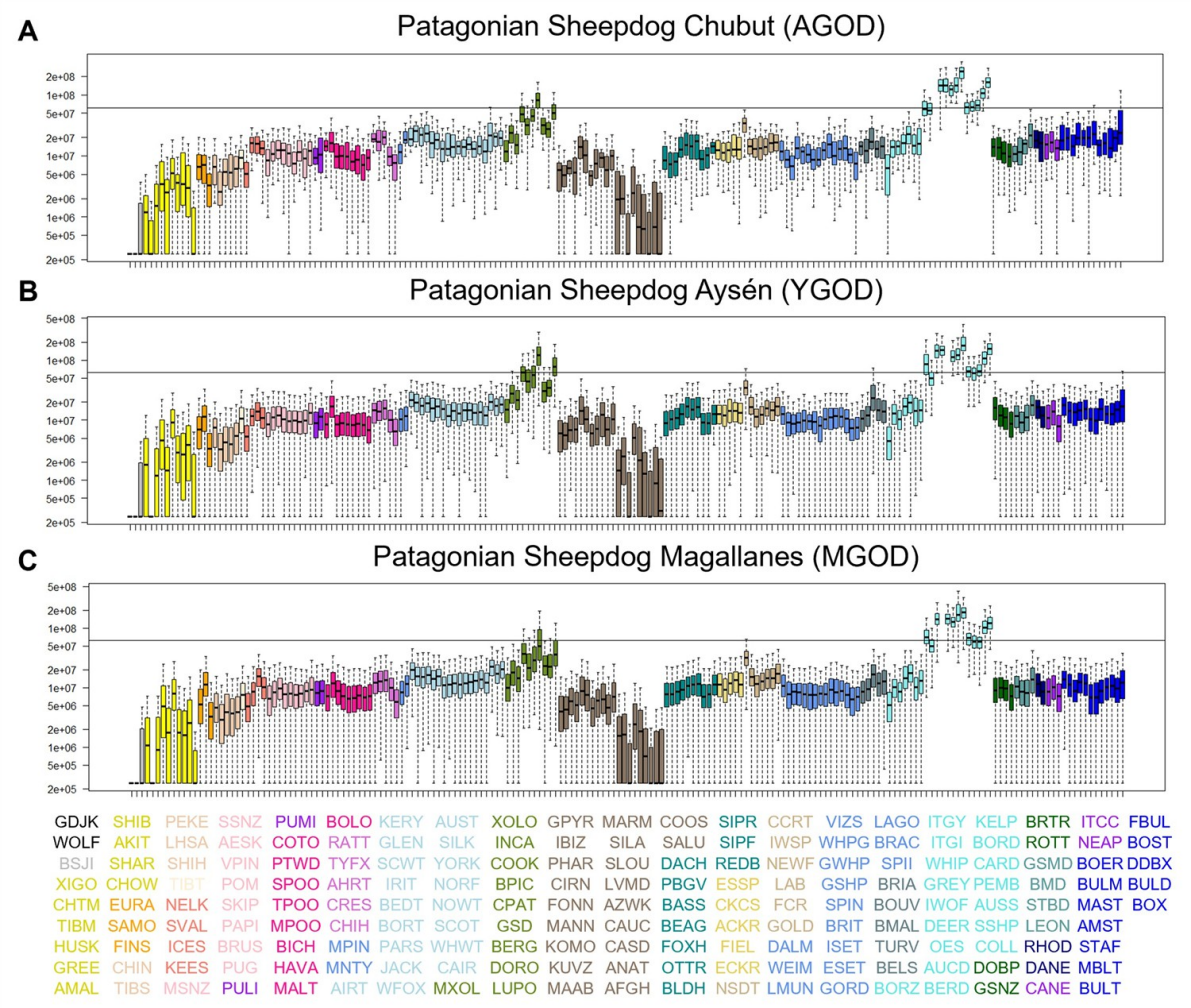
Haplotype sharing analyses among 176 breeds, including PGODs from three Patagonian regions. Haplotype sharing between the PGOD populations of AGOD (A), YGOD (B), and MGOD (C) and 175 breeds. Each boxplot represents haplotypes shared between the PGOD and the each of the 175 non-PGOD breeds. Analyses used a window size of 1,000 SNPs with an overlap of 25 SNPs. Dog breed abbreviations are positioned and colored on the x-axis. Each color represents a group of breeds that belong to a previously defined clade [5]. Breeds that represent the UK rural clade are shown in light blue. The horizontal line indicates the level below which 95% of breed pairs from different clades share haplotypes. Breeds with median values above this line are determined to have significant haplotype sharing with the breed being analyzed, in this case, the specific PGOD populations. Breed abbreviations can be found in S1 Table.

To better understand the relationship between PGODs and the other breeds included in the phylogeny (Fig 2), we analyzed genomic similarity using identity by descent (IBD) methods to identify shared haplotypes among 176 dog breeds assigned to 23 clades (Fig 3). In this analysis, PGODs were divided into three groups based on their geographic sampling location: (i) Chubut, Argentina (AGOD), (ii) Aysén, Chile (YGOD), both located in northern Patagonia, and (iii) Magallanes, Chile (MGOD) in southern Patagonia. All three populations show significant levels of haplotype sharing with all herding breeds of the UK rural clade, except for the Australian cattle dog. Additionally, gene flow from German shepherd dog (GSD), a breed from the New World clade, to YGOD and AGOD populations was observed (Fig 3A and 3B and S2 Table). Haplotype sharing between the YGOD and Lupo italiano (LUPO) also reaches significance. However, we hypothesize that, rather than a recent admixture event in Patagonia between PGOD and LUPO, these results reflect the previously defined historical relationship between GSD and LUPO [4]. Based on these results we generated a new dataset with 11 breeds, nine from the UK rural clade plus the GSD and the PGOD. This dataset, with 247 dogs in total, is hereafter referred to as the “herding dog subset” (S1 Table).

### Population structure

**Fig 4:**
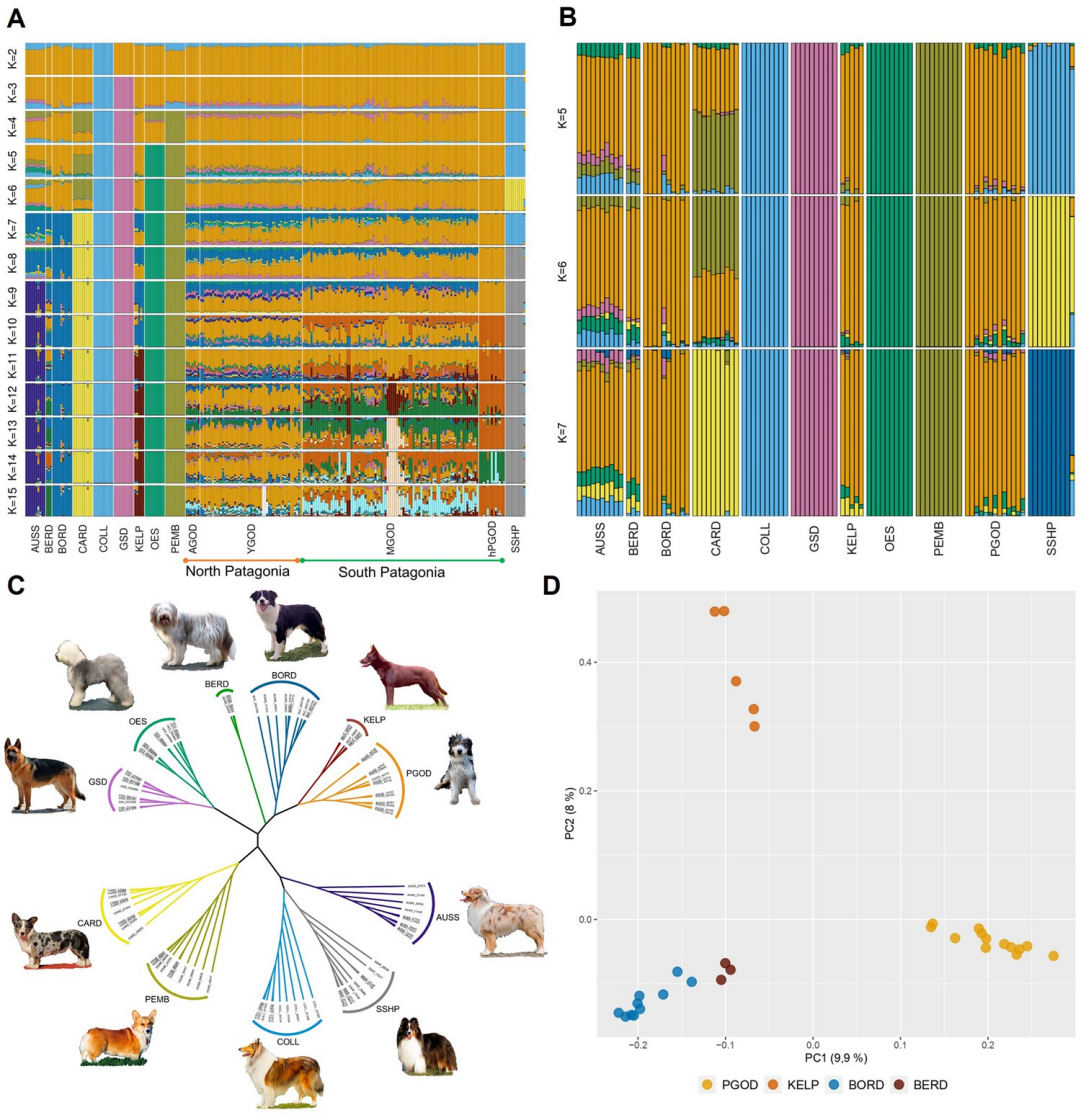
Genomic structure and ancestry composition analyses. (A) Admixture analyses performed between the eleven breeds of the herding dog subset which includes Patagonian sheepdog (PGOD) from Chubut, Argentina (AGOD), Aysén (YGOD), Magallanes (MGOD), the 13 homogenous PGOD individuals from Magallanes (hPGOD), Australian shepherd (AUSS), Bearded collie (BERD), Border collie (BORD), Cardigan welsh (CARD), collie (COLL), German shepherd (GSD), Australian kelpie (KELP), old English sheepdog (OES), Pembroke welsh (PEMB) and Shetland sheepdog (SSHP). The proportion of ancestry for each individual is shown, assuming 2 to 15 ancestral populations (K). Maximum likelihood predicts the grouping of K = 8 (determined by ADMIXTURE’s cross-validation procedure) as the probable number of dog populations. (B) Population structure for 13 homogenous PGODs and ten breeds comprising the herding subset. Each column represents a single dog. Maximum likelihood predicts the grouping of K = 6 as the optimal number of dog populations. (C) An unrooted neighbor-joining tree indicates the relationships between ten breeds of the herding dog subset (247 dogs) and the homogenous PGODs. (D) Principal Component Analysis (PCA) plot of PC1 and PC2, shows the distinct separation of the homogenous PGOD from BORD, BERD, and KELP. Each dot represents an individual and different colors indicate different breeds.

To better understand the genomic composition of the PGOD, we explored the population structure and degree of admixture between the PGOD and the ten breeds in the herding dog subset (Fig 3). We tested values of K ranging from 1 to 15, where K is the assumed number of populations [18]. The analysis showed the lowest cross validation (CV) error (0.57) (S1A Fig) for K = 8, suggesting eight as the most likely number of genetically distinct groups within the dataset. The plots obtained for the expected number of populations, K = 2–15, are shown in Fig 4A, with K = 15 being the number of assumed populations that allowed separation of each registered breed into its own cluster.

The admixture models illustrate the degree of diversity and variability within the PGOD population, which shows greater evidence of genetic heterogeneity compared to other herding breeds, as reflected by the continued mixed composition through increasing values of K (Fig 4A). Furthermore, a clear geographic differentiation in genomic structure among the PGODs was observed. Comparing the genomic structure between PGOD populations for K = 8, we observed that the northern populations are comprised of genomic signatures consistent with PGOD, BORD, GSD and OES at levels of 41% (SD = 4%), 32% (SD = 3%), 9% (SD = 3%) and 7% (SD = 2%) respectively, for AGOD; while the levels are 42% (SD = 6%), 31% (SD = 8%), 9% (SD = 3%) and 8% (SD = 3%) respectively, for YGOD. Comparatively, the southern MGOD population is comprised of the same breed signatures but at levels of 72% (SD = 15%), 15% (SD = 8%), 5% (SD = 6%) and 3% (SD = 3%), respectively. These geographically-associated differences suggest that the southern population of PGODs shows overall a greater proportion of the genomic signature specific to PGODs (mean = 72%, SD = 15%) but greater variability in the minor ancestry components. In comparison, the population of northern Patagonia shows lower levels of the initial PGOD component (mean = 41%, SD = 4% for AGOD; and mean = 42%, SD = 6% for YGOD), but a more consistent pattern of component signatures among individuals.

Analysis of the ancestry composition of each individual PGOD, a group of 13 dogs from Tierra del Fuego, an island in southern Patagonia, showed very limited contribution from minor ancestry components (over 95% PGOD ancestry) relative to PGODs collected from the mainland of southern Patagonia (Fig 4A). We speculate that this subset of PGODs may represent a more historically accurate version of the breed, hence, we repeated the ADMIXTURE analysis using only the 13 most homogenous PGODs (S5 Table), hereafter referred to as the “homogenous PGODs” (Fig 4B), and the herding breed subset. We tested values of K ranging from 1 to 12. The lowest CV error (0.60) (S1B Fig) was obtained for K = 6 (Fig 4B). Interestingly, this new admixture analysis identifies a common genomic signature prevalent in the PGOD, KELP, and BORD populations (92%, 91% and 95%, respectively), suggesting that these breeds all descend from the same ancestral population. This signature is also seen at 73% in BERD and 65% in AUSS (Fig 4B).

An unrooted neighbor-joining tree was also built to demonstrate the relationship between the herding breed subset and the homogenous PGODs. The homogenous PGODs are monophyletic with the BORD, BERD, and KELP (Fig 4C). A PCA was generated to examine the relationship of these homogenous PGODs with BORD, BERD, and KELP (Fig 4D). The first two principal components (PCs) explain 9.9% and 8% of the total genetic variance between these closely related breeds, respectively. The first principal component (PC1) produced a major separation of PGOD from the rest of breeds, while PC2 separated KELP from BORD, BERD, and PGOD.

### Effective population size and estimation of migration events

The effective population size (N_e_) of each herding breed was estimated through SNP-based linkage disequilibrium (LD) analysis, considering a timeframe of 13 to 150 generations. To better represent the N_e_, PGODs were separated by region. Seven AGODs were available, which shows an N_e_ of 30 at 13 generations, and 308 individuals at 150 generations. Random subsets of 10 dogs each from YGOD and MGOD were generated to calculate N_e_ statistics (S3 Table). At 13 generations a mean (range) N_e_ of 48 (47–49) individuals for YGOD and 47 (44–53) for MGOD were observed. While at 150 generations a mean (range) N_e_ of 446 (437–451) individuals for YGOD and 419 (394–456) for MGOD was observed (S3 Table). The next comparable breeds are BORD and AUSS, showing an N_e_ of 312 individuals at 150 generations (S3 Table). The effective population size estimation suggests a larger ancestral pool for PGOD compared to the other herding breeds, which is independent of the region of origin. This original population size is larger than the current population of PGODs, likely reflecting the changing need for sheepdogs in the region.

Potential migration events and gene flow between PGOD and the other ten breeds from the herding breed subset were investigated using the software Treemix v.1.12 [19]. Two maximum likelihood trees of the population without migration events and using the golden jackal as an outgroup were performed for region-specific PGODs and for the homogenous PGOD group. We then incorporated events sequentially up to 10 migrations. The optimal number of migrations was estimated with the *optM* R package (S2 Fig). We determined that the most probable migratory events were m = 3 and m = 2, and we plotted the residual matrices for PGOD separated by regions and the homogenous PGOD group (S3 Fig). When analyzing PGOD separately by geographic origin, MGOD, YGOD and AGOD were placed on the same branch as BORD, KELP and BERD, and showed gene flow from GSD to both AGOD and YGOD in two migration events (S3A Fig). Interestingly, when we analyzed the group of homogenous PGODs, the algorithm again placed PGOD in the same branch as BORD, KELP and BERD, but also suggested gene flow from a common ancestor between UK herding dogs and GSD to PGOD in one migration event (S3B Fig). The proposed GSD admixture events were corroborated by D-statistics analysis (S2 Table).

### Genome diversity

**Fig 5:**
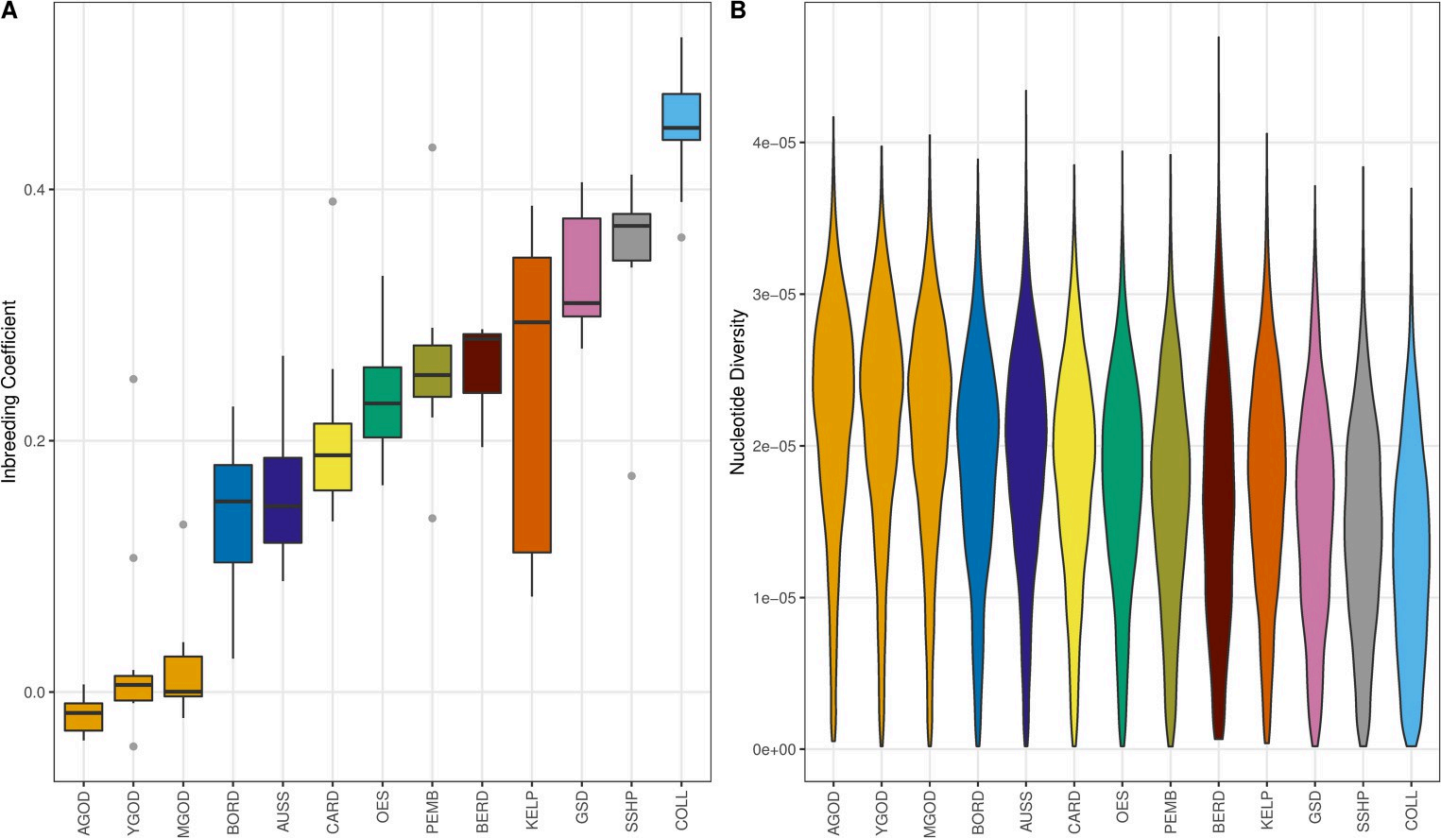
Genome diversity analyses. (A) Boxplot for coefficient of inbreeding calculated using SNP data from the herding dog subset which includes Patagonian Sheepdog (PGOD) from Chubut, Argentina (AGOD), Aysén (YGOD), Magallanes (MGOD), Border collie (BORD), Australian shepherd (AUSS), Cardigan welsh (CARD), old English sheepdog (OES), Pembroke welsh (PEMB), Bearded collie (BERD), Australian kelpie (KELP), German shepherd (GSD), Shetland sheepdog (SSHP) and collie (COLL). Horizontal lines indicate the median, and lower and upper hinges which correspond to the 25th and 75th percentiles. Outlier points are shown. (B) Distribution of nucleotide diversity in 500-kb non-overlapping windows across herding breeds.

We estimated inbreeding coefficients in the breeds from the herding dog subset using PLINK v1.9 [20]. PGODs were separated by geographic region. We selected random groups of 10 individuals for YGOD and MGOD for the calculations, and one of these groups was randomly chosen for the graph (see “Materials and Methods”, S4 Table). All groups of PGODs demonstrate lower levels of inbreeding when compared to the other herding breeds. The lowest mean inbreeding coefficients were observed as follows: AGOD: -0.02; MGOD: 0.02; and YGOD: 0.03 (S5 Table), while the highest was observed in COLL (0.45) (Fig 5A). Similar values were obtained when analyzing all PGODs in a single group (n = 159), where the inbreeding coefficients ranged from -0.04 to 0.27 with a mean of 0.03 (S6 Table). We also calculated nucleotide diversity as it provides an estimate of polymorphism within populations. Analysis was performed in 500-kb non-overlapping windows [21]. Again, random groups of 10 individuals were constructed for YGOD and MGOD and one group was randomly selected for the graph. Compared to the other herding dogs, the three PGOD populations showed the highest levels of nucleotide diversity (Fig 5B). Altogether, these results suggest that PGOD has the highest genetic diversity compared to the other herding breeds considered in this study.

### Estimated date of herding breed divergence

**Fig 6:**
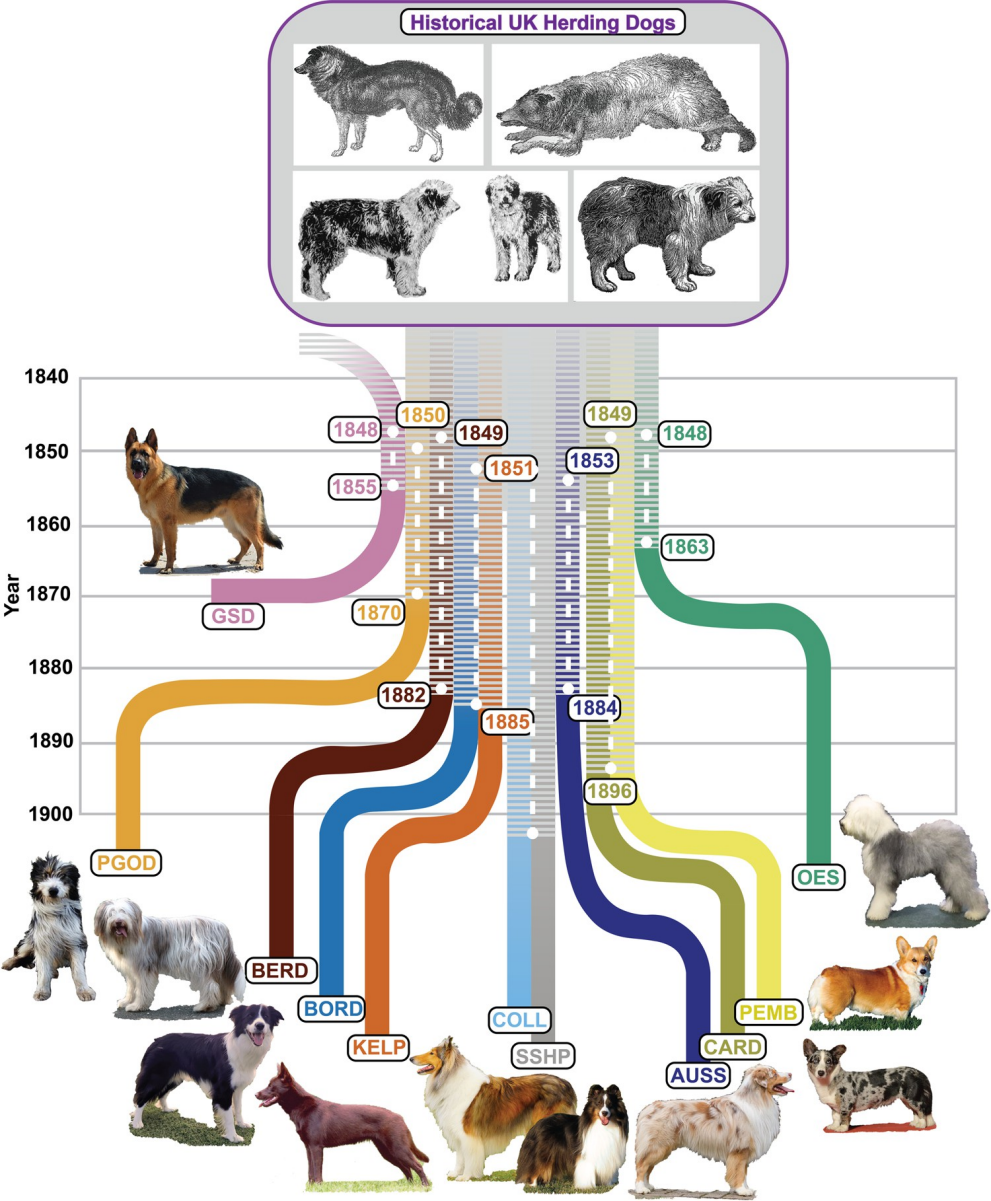
Estimated date of herding breed divergence. The timeline representation shows the estimated years of genomic divergence of each UK herding breed from a theoretical common foundational population. The breeds considered here were Patagonian Sheepdog (PGOD), Bearded collie (BERD), Border collie (BORD), Australian kelpie (KELP), collie (COLL), Shetland sheepdog (SSHP), Australian shepherd (AUSS), Cardigan welsh (CARD), Pembroke welsh (PEMB), and old English sheepdog (OES). Black and white images depicting types of British sheepdogs in publication from 1845 (top) and 1879–1899 (bottom) [22–24]. While the German shepherd (GSD) is not considered part of the UK herding clade, it shows most recent haplotype sharing with the UK breeds between 1848 and 1855. White dashed lines indicate divergence date ranges calculated from the median length of haplotype sharing for each breed with the remaining UK herding breeds. The PGOD shows breed-to-breed divergence dates of between 1850 and 1870, prior to the genetic separation of most of the UK herding breeds. The COLL and SSHP do not show genetic divergence in this representation due to the limitations of predicting more recent divergence events with this dataset.

As published previously, we adjusted a linear model of the relationship between the total length of shared haplotypes and the historical date of an admixture or divergence event, using nine pairs of breeds [5]. We estimated the slope and intercept that describe the relationship and used it to estimate the year of genomic divergence between each pair of breeds from the herding dog subset using the 13 homogenous PGODs (S7 Table). The PGOD samples utilized here were obtained in 2019 so the estimated years of divergence are considered relative to this date. The divergence dates of the PGOD with each UK herding breed are ~149 years ago, whereas the divergence dates of the non-PGOD breed pairs are calculated to have occurred more recently [22–24] (Fig 6).

## Discussion

The genomic characterization of rare regional dog populations has become a powerful tool for uncovering the demographic history of such populations, and can also be used to track the movement of human populations in the same regions [1,6,25,26]. The historical circumstances that prompted the migration of humans and their herding dogs from the UK to Patagonia in the mid-1800’s, and the resulting genetic isolation of these populations, presents a valuable opportunity to explore the genetic implications of this shared journey.

In this study, we explored the relationship of PGODs with other dog breeds through phylogenetic and haplotype sharing analyses. We combined genome-wide marker data from 159 PGODs with genomic information from 1514 individuals representing 175 dog breeds and two wild canids published previously [4,5]. The phylogeny obtained assigned all 159 PGOD individuals to the previously defined UK rural breed clade [5]. However, they did not cluster as a single population (Fig 2). The relationship of PGOD with the UK rural clade is explained by the European migration history throughout Patagonia, with the initial arrival of three hundred sheep from the Falkland Islands, a UK territory, to the Magallanes region of Chile in 1877 [12,13]. This was followed by co-migration of people and sheep from the Falklands to Magallanes during the 1880s (Fig 1A). The colonization incorporated both techniques and standards used by traditional Anglo-Scottish herders, which are still maintained today in Southern Patagonia [9,13].

The migration of shepherds and their dogs continued towards the Aysén region in the Northern Chilean Patagonia through Argentina [9,27]. This historical migration route (Falklands—Magallanes—Argentina/Aysén) is reflected in the current geographic distribution of PGODs and their phylogeny, where two well-defined subpopulations of PGODs are identified, separated by their location in Magallanes and Aysén/Chubut to southern and northern Patagonia, respectively (Fig 2). The two PGOD subpopulations are separated by natural geographic barriers, the most significant being ice fields (Fig 1A). Despite their geographical separation, PGODs from both subpopulations are strikingly similar in appearance and behavior (Fig 1D), likely demonstrating selection for a common function. Validation of this historical selection can be found in documents related to the recruitment of workers from Scotland, where it was stipulated that a sheepherder had to bring with him one or two sheep dogs. Those with pups, signifying the potential for the dogs to reproduce, would receive preference for recruitment [9,28]. These early historical records describe an early settlement, for which human-animal partnerships were key to the success of pastoral activities.

Although phylogenetic trees can describe the relationship between breeds, they cannot provide a full understanding of breed development and evolution [29]. Therefore, to determine the placement of PGOD within the UK rural clade we analyzed hybridization across clades, looking for shared haplotypes between breeds. Despite the geographic isolation that has impacted the PGOD, all three subpopulations share haplotypes with the same nine herding breeds from the UK rural clade (Fig 3), inferring that the exchange of haplotypes occurred before the arrival of PGOD to South America and its separation throughout Patagonia.

The dog breeds of the collie lineage share the same ancestors as working sheepdogs from Great Britain before the explosion of modern breeds [2]. Among breeds likely related to PGOD before and during the period of extensive Patagonian colonization, between 1877–1910, are the Old Welsh Grey and other historic UK herding dogs which have similar aptitudes for herding sheep [9,15]. However, these regional varieties disappeared from the UK at the end of the 19^th^ century as a result of industrialization and changes in trade and transportation patterns [9,14].

To further disentangle the relationship between PGOD and the UK herding breeds, we examined ancestry patterns using the clustering software Admixture (Fig 4A). The PGOD showed higher genetic heterogeneity compared to other herding breeds. Also, within PGOD, the MGOD subpopulation shows a smaller contribution from other breeds to its ancestry composition, likely due to their greater degree of geographic isolation. Most dogs from Southern Patagonia inhabit the island of Tierra del Fuego, which acts as a geographic barrier, reducing admixture with other breeds. Also, sub-structure within the PGOD provides evidence at the local level that the individuals from southern Patagonia have been distinct from YGOD and AGOD for long enough to acquire distinguishable genetic identities between them. When compared to MGOD, the ancestry composition of the AGOD and YGOD shows a greater contribution of BORD, likely due to recent admixture after BORD’s introduction to Patagonia over the last 15 years.

It is possible that PGOD sub-populations would have experienced different levels of genetic drift, whereas the MGOD population seems to have been subjected to greater drift, as shown by the 13 dogs defined as "homogenous PGODs". Although, these dogs show a higher inbreeding coefficient than other PGOD sub-populations, the “homogenous PGODs” shows less inbreeding than the modern herding breeds analyzed here (S5 Table). This suggest that the bottlenecks experienced by modern herding breeds is stronger than those caused by the migration event of PGOD to Patagonia, and the subsequent local breeding and selection scheme.

When we explore the genomic structure using the homogenous PGODs from Magallanes, we observe that admixture analysis is not able to separate the PGOD, KELP, and BORD populations. The similar ancestry pattern exhibited by these breeds likely stems from shared common ancestry rather than from recent admixture (Fig 4B).

Interestingly, a strong genetic signature from the PGOD/KELP/BORD group to the BERD and AUSS was observed (73% and 65%, respectively) (Fig 4B), providing further evidence of a common genetic signature shared across all herding breeds analyzed here. The close genetic relationship between these herding breeds probably occurred before the middle of the 19th century when the different types of herding dogs were not yet separated into distinct breeds, and breeding by shepherds focused solely on obtaining an intuitive and intelligent dog, independent of its pedigree. This system, with no specific selection for any morphologic traits, gave rise to a great diversity of coat type and color in herding dogs. However, particular lines developed in some regions, establishing themselves as local or niche breeds whose behaviors, ability to learn, and perhaps even morphologic features made them ideal for a particular terrain, weather pattern, or stock type [23].

In 1875 two broad types of sheepdog varieties existed: rough-coated and smooth-coated [30]. Subsequently, publications dated between 1885 and 1890 describe three different British varieties: the Scottish collie or rough-haired sheepdog, the smooth-haired sheepdog, and the old English short-tailed sheepdog [31]. These simple descriptions highlight the absence of formalized breed structure among herding dogs in the mid-1800’s in the UK. It is not until the late 1800’s that official breed clubs were formed for the select purpose of producing standardized dog breeds. These population splits are consistent with the haplotype sharing data from our IBD analyses, wherein the modern UK herding breeds show breed-to-breed divergence dates between 1863 and 1896. Conversely, the haplotype-derived divergence points of the PGOD from each of the modern herding breeds dates to between 1850 and 1870, overlapping with the known migration timeframe of herding dogs to Patagonia and occurring prior to the formation of the modern UK herding breeds (Fig 6). Indeed, the recorded breed origins of the modern UK herding breeds correspond to dates within 10–30 years of our IBD-derived divergence dates (Table 1). Further, analysis of human genetic admixture involving the native populations of Chilean and Argentinian Patagonia indicate a post-colonization influx of European ancestry, specifically from Great Britain that dates to between 1763 and 1931 [32,33]. This once again highlights the genomic patterns observable due to the parallel migration of humans and their dogs.

**Table 1:** IBD haplotype-derived dates of the most recent breed divergence of UK herding breeds relative to the historical records of breed development.

Breed	Genetic Divergence Date	Historical Date of Breed Origin	Historical Reference
AUSS	1853–1884	Late 1800’s	importation of ancestors [34]
BERD	1849–1882	1912	breed standard [35]
BORD	1851–1885	1893	common ancestor [36]
CARD	1849–1896	1925	dog show records [37]
COLL^a^	1851–2012	1860’s	dog show records [38]
KELP	1849–1885	1875	importation of ancestors [39,40]
OES	1848–1863	1885	breed description [31]
PEMB	1849–1896	1925	see CARD
PGOD	1850–1870	1877	importation of ancestors [9]
SSHP^a^	1850–2012	1906	dog show records [41]

^a^ The 2012 date is estimated based on haplotype sharing between COLL and SSHP. This method of divergence date estimation may be inaccurate for closely related breeds, such as the COLL and SSHP, due to known modern introgressions, common phenotypes, and similar selection trajectories.

Our ADMIXTURE analysis revealed a genomic structure in the modern PGOD that is very similar to the genomic structure of BORD, BERD, and KELP. While these breeds are readily distinguishable through our other genetic analyses, this common genomic structure likely reflects the remnant influence of the old working sheep dogs of Great Britain (Fig 4B). Interestingly, the ADMIXTURE analysis shows a low contribution from GSD to the genomic structure of most PGODs (9% and 5% of ancestry in AGOD-YGOD and MGOD, respectively). This finding is reinforced by the Treemix analysis, where maximum likelihood trees inferred migration events from the GSD to AGOD and YGOD (S3A Fig). A migration event from a common ancestor between the UK herding breeds and GSD to the homogenous PGODs was also identified (S3B Fig). It is not surprising that the GSD, which was previously assigned to a group of breeds classified as the New World clade [5], could have a shared genetic history with the UK herding breeds. Previous studies demonstrate significant haplotype sharing among Italian herding breeds and GSD, with admixture/divergence events between 1859–1867 [4]. This suggests that herding behavior has arisen from multiple geographic and genetic backgrounds [5]. The influence of a pervasive common livestock dog from continental Europe, from which the GSD originated, has left an agrarian signature in many breeds [4], including PGOD, with which it has a divergence date of approximately 1850 (Figs 6 and S3B).

Genetic variability and the structure of a domestic breed depends largely on the breeders’ decisions and practices [42]. When we analyzed PGOD’s inbreeding coefficient and nucleotide diversity, the three PGOD populations showed a higher level of genetic diversity than other breeds from the herding dog subset analyzed here (Fig 5). This agrees with PGOD’s higher effective population size, which indicates that low levels of genetic drift have occurred in the PGOD population, allowing it to maintain high genetic diversity and a low level of inbreeding compared to other herding breeds. Indeed, we obtained a mean N_e_ of 308 individuals for AGOD, 446 for YGOD, and 419 for MGOD at 200 to 300 generations before present, which exceeds the suggested minimum population size of 50 to 100 individuals for the establishment and recognition of a breed (http://www.fci.be/en/Standing-Orders-of-the-FCI-40.html). The higher genetic diversity and N_e_ in PGOD may be explained by the fact that although these dogs are strongly selected for success in a pastoral environment; they are not under selection to conform to aesthetic standards [9]. Similar findings were described in endemic dog landrace populations from Italy [43].

To genetically characterize a landrace dog breed from Patagonia, our analyses also identified a lost reservoir of ancestral dogs reflecting the foundational population that ultimately gave rise to the modern UK herding breeds. Genomic analyses, coupled with historical documentation trace the origin of the PGOD to the UK, prior to the explosion of modern breeds in the Victorian era. The PGOD belongs to the clade of dogs that share UK heritage, typified by the modern BORD, BERD, and KELP breeds. We propose that the PGOD is the closest living representative of the common ancestor to the original UK herding breeds, mirroring how the foundational UK sheepdog looked and performed, and displaying skills that are preserved by the PGOD in modern Patagonia.

## Materials and methods

### Ethics statement

The field sampling and study protocols were conducted and approved according to the Universidad Austral de Chile Institutional Animal Ethics Committee (N° 317/2018) and reviewed and approved by ANID-Chile (Agencia Nacional de Investigación y Desarrollo, Chile). The sample collection was authorized by owners with a signed consent, in accordance with National Human Genome Research Institute (NHGRI), Animal Care and Use Committee protocols.

### Dog samples and DNA extraction

Whole blood samples were obtained from 159 Patagonian sheepdogs (PGOD). Based on owner knowledge, the sampled PGODs were unrelated to at least three generations within a sampling location. The sampled PGOD come from three provinces of the Southern Chilean regions of Magallanes and Antarctica (Magallanes, Tierra del Fuego, and Última Esperanza (n = 101)), four provinces of the Northern Aysén region (Coyhaique, Aysén, General Carrera and Capitán Prat (n = 51)), and Chubut province, Argentina (n = 7) (Fig 2B). Blood samples were collected by veterinarians through venipuncture of the cephalic vein (3 to 5 ml) from working dogs that comply with the Patagonian sheepdogs morphometric standards [15,44]. Blood samples were collected in acid citrate dextrose anticoagulant (ACD) tubes. Samples were stored at 4°C prior DNA extraction, and extraction was performed for all blood samples using standard proteinase K/phenol-chloroform isolation methods. Finally, samples were stripped of identifiers, numerically coded, suspended in TE (10 mM Tris base, 0.01 mM EDTA), aliquoted and stored at -80°C.

### Data collection

A set of 159 PGODs was genotyped using the Illumina CanineHD Whole-Genome Genotyping BeadChip (San Diego, CA, USA), which has 173,662 potentially informative markers. This process was carried out at the National Human Genome Research Institute (NHGRI) of the National Institutes of Health (Bethesda, MD, USA). Genotype calls were conducted in Illumina Genome Studio, specifying a 90% call rate. In addition, genotyped samples were merged with a dataset of 175 breeds generated for a previous studies [4,5]. The final dataset included 150,069 SNPs.

### Phylogenetic and genetic distances estimation

PLINK v1.9 [20] was used to calculate genetic distances between 1673 individuals, representing 176 breeds, including the PGOD population and two wild canids. The genetic distance was estimated using the “—distance” and the “1-ibs,” “square,” and “flat-missing” modifiers [20]. Neighbor-joining phylogeny and consensus tree calculation was built using the PHYLIP software package v3.698 [17] with 100 bootstraps, and golden jackal (GDJK) as an outgroup. The bootstrapped cladogram of the consensus tree was drawn in FigTree v.1.4.4 [45]. Breeds were assigned to clades and light blue was used to represent the UK rural clade, relative to the expected clade structure published previously [5]. The unrooted tree was built with the same method detailed above using the herding dog dataset (247 dogs from 11 breeds), but without the outgroup.

### Shared haplotypes

Haplotype sharing was determined by identity-by-descent (IBD) estimations among individuals. This analysis was performed on the 176 dog breeds and two wild canids with Beagle v4.1 [46]. The dataset was analyzed in windows of 1,000 SNPs with an overlap of 25 SNPs. Haplotype sharing was considered significant when median values fell above the 95^th^ percentile of all across-clade breed pairs. Boxplots of haplotype sharing distributions between breeds were performed using R Core Team [47].

### Population structure of patagonian sheepdogs

The genetic structure and the extent of admixture between PGOD and related herding breeds was evaluated through the model-based clustering algorithm implemented in the ADMIXTURE software v1.3 [18]. To reduce prediction error, ADMIXTURE cross- validation (CV) was used to determine the optimal K-value, minimizing the CV error using the script described in the ADMIXTURE documentation [18]. K represents the number of populations assigned during each clustering run. To assess the population structure of the herding breeds including PGOD, we ran a PCA using the R package *flashpcaR* [48]. Gene flow was calculated by D-statistics using the R package ADMIXTOOLS [49].

### Effective population size and estimation of migration events

To calculate the effective population size of herding dogs we used SNeP v1.1 [50]. PGODs were separated by geographic region. Because of the uneven sample size per region, we randomly selected (without replacement) groups of 10 individuals for YGOD and MGOD by using the sample function from base package in R. All AGOD individuals available were used for the calculations on this group.

The herding dog dataset was separated into unique files by breed and the “ped” and “map” files were created with the parameter “-recode12” in PLINK software v1.9. A maximum likelihood tree was constructed with Treemix v.1.12 [19]. The trees were produced by analyzing the data in windows of 1,000 SNPs using the flags -k 1000 and 1,000 bootstrap repeats using the flag -bootstrap 1000 parameter and allowing for one to ten migration events. To estimate the number of migration edges on a population tree we use the R package *optM* (https://cran.r-project.org/web/packages/OptM/index.html). We ran five iterations at each migration event as *optM* requires at least two iterations to be run for each value of the number of migration events (m value).

### Intra- and inter-breed genomic diversity

Inbreeding coefficients (F) were calculated from 247 dogs from 11 herding breeds using the “—het” function of PLINK v.1.9 [20]. The maximum, minimum, and mean values were obtained. The breed-specific F value was determined by averaging individual F values for all dogs of a single breed. Nucleotide diversity was estimated in 500-kb non-overlapping windows with the VCFTools software v0.1.15, using the parameters: window_pi. [21]. As explained in the effective population size analysis, PGODs were separated by geographic region and the sample function in R was used for the random selection procedure. These results were graphed using the R package ggplot2 [47,51].

### Estimated date of herding breed divergence

To calculate the number of years since shared genetic history observed between the herding breeds analyzed here, we used a linear model between the total length of haplotype sharing and the age of a known admixture or divergence event, occurring between 35 and 160 years before present [5]. We adjusted the model using nine pairs of breeds and applied this equation to the total shared haplotypes calculated from the genotyping data. We estimated the slope and intercept that describe the relationship and used it to estimate the year of genomic divergence between each pair of breeds (linear correlation r^2^ = 1). We used the relationship equation y = -8736150x + 1501072917 on our herding data set, including the 13 homogenous PGODs, to determine historical time estimations, where y is the total shared haplotype length and x is the number of years. The PGOD samples utilized here were obtained in 2019 so the estimated years of divergence are considered relative to this date.

## Supporting information

S1 Table Breed abbreviation. Abbreviation of 176 breeds including the Patagonian Sheepdog (PGOD) separated in three regions: Chubut, Argentina (AGOD), Aysén, Chile (YGOD), and Magallanes, Chile (MGOD), and two wild canids. N is the number of individuals for each breed. Summary of breed abbreviations corresponding to Figs 2 and 3.
(XLSX)

S2 Table Gene flow signatures from D-statistics test. (XLSX)

S3 Table Effective population size (N_e_) of herding dogs. N_e_ was obtained based on the calculation of linkage disequilibrium (LD) between SNP markers considering a timeframe of 13 to 150 generations ago. PGOD was separated by region of origin in AGOD, YGOD and MGOD. Random subsets of 10 dogs from YGOD and MGOD were generated to calculate N_e_. Table shows mean, minimum and maximum range of YGOD and MGOD random groups.
(XLSX)

S4 Table Inbreeding coefficients of Patagonian sheepdogs. Inbreeding coefficients of PGOD (mean, minimum and maximum values) calculated in random samples of 10 to 11 dogs.
(XLSX)

S5 Table Inbreeding coefficients of herding dogs. PGOD were separated by region and a random sample of 10 ~11 individuals were used to calculate inbreeding coefficients for MGOD and YGOD.
(XLSX)

S6 Table Inbreeding coefficients between herding dogs and Patagonian sheepdogs. All sampled PGODs were used to calculate the inbreeding coefficient.
(XLSX)

S7 Table Estimated date of herding breed divergence. Haplotypes shared between each pair of herding breeds, considering the 13 homogenous PGOD.
(XLSX)

S1 Fig Cross validation (CV) error plot for ADMIXTURE analysis. Line graph of CV error values for each ancestry models denoted by K. The upper plot (A) shows the CV error for herding related PGOD dogs, the red dot is the minimal CV error (0.57020). The bottom plot (B) shows the CV error for homogenous PGOD dataset, the blue dot is the minimal CV error (0.59766).
(TIF)

S2 Fig Line plot of optimal number of migration edges. Line plot of the optimal number of migration edges on each population calculated through optM with Treemix output. (A) optM output using Treemix results of subset herding dogs. (B) optM output using Treemix results of subset considering the homogenous PGOD dogs.
(TIF)

S3 Fig Maximum likelihood tree of the inferred relationships between herding dog breeds. Maximum likelihood trees show the most important migration events. Scale bar shows ten times the average standard error of the sample covariance matrix. The estimated migration between breeds and gene flow are shown according to by direction and weight (yellow to red = 0 to 0.5). (A) Maximum likelihood tree using three migration events within the herding dog subset and PGODs separated by region in AGOD, YGOD, and MGOD. The residual matrix is plotted from a TreeMix analysis under 3 migration events (m = 3). (B) Tree using two migration events within the herding dog subset and the homogenous PGODs. The residual matrix is plotted from a TreeMix analysis under 2 migration events (m = 2). The breed abbreviations correspond to S1 Table.
(TIF)

## References

[1] Ostrander EA, Wayne RK, Freedman AH, Davis BW. Demographic history, selection and functional diversity of the canine genome. Nat Rev Genet. 2017;18: 705–720. doi: 10.1038/nrg.2017.67 28944780

[2] Worboys M., Strange J.M. PN. The invention of the modern dog: breed and blood in Victorian Europe. 1st ed. Baltimore: Johns Hopking University Press; 2018.

[3] Plassais J, Kim J, Davis BW, Karyadi DM, Hogan AN, Harris AC, et al. Whole genome sequencing of canids reveals genomic regions under selection and variants influencing morphology. Nat Commun. 2019;10: 1–14. doi: 10.1038/s41467-018-07882-8 30940804 PMC6445083

[4] Talenti A, Dreger DL, Frattini S, Polli M, Marelli S, Harris AC, et al. Studies of modern Italian dog populations reveal multiple patterns for domestic breed evolution. Ecol Evol. 2018;8: 2911–2925. doi: 10.1002/ece3.3842 29531705 PMC5838073

[5] Parker HG, Dreger DL, Rimbault M, Davis BW, Mullen AB, Carpintero-Ramirez G, et al. Genomic analyses reveal the influence of geographic origin, migration, and hybridization on modern dog breed development. Cell Rep. 2017;19: 697–708. doi: 10.1016/j.celrep.2017.03.079 28445722 PMC5492993

[6] Dreger DL, Davis BW, Cocco R, Sechi S, Di Cerbo A, Parker HG, et al. Commonalities in development of pure breeds and population isolates revealed in the genome of the Sardinian Fonni’s dog. Genetics. 2016;204: 737–755. doi: 10.1534/genetics.116.192427 27519604 PMC5068859

[7] Spady TC, Ostrander EA. Canine behavioral genetics: pointing out the phenotypes and herding up the genes. Am J Hum Genet. 2008;82: 10–18. doi: 10.1016/j.ajhg.2007.12.001 18179880 PMC2253978

[8] Bergström A, Frantz L, Schmidt R, Ersmark E, Lebrasseur O, Girdland-Flink L, et al. Origins and genetic legacy of prehistoric dogs. Science (80-). 2020;370: 557–564. doi: 10.1126/science.aba9572 33122379 PMC7116352

[9] Barrios N, Fuenzalida A, Gómez M, Heuser C, Muñoz R, Ostrander EA, et al. The Patagonian sheepdog: historical perspective on a herding dog in Chile. Diversity. 2019;11: 1–12. doi: 10.3390/d11060087 34712100 PMC8549857

[10] Gajaweera C, Kang JM, Lee DH, Lee SH, Kim YK, Wijayananda HI, et al. Genetic diversity and population structure of the Sapsaree, a native Korean dog breed. BMC Genet. 2019;20: 1–11. doi: 10.1186/s12863-018-0706-8 31382890 PMC6683530

[11] Martinic M. Presencia de Chile en la Patagonia austral 1843–1879. 1st ed. Santiago: Andrés Bello Press; 1971.

[12] Martinic M. Breve Historia de Magallanes. 1st ed. Punta Arenas: Universidad de Magallanes Press; 2002.

[13] Martinic M. Los Británicos en la Región Magallánica. 1st ed. Valparaíso: Universidad de Playa Ancha Press; 2007.

[14] Combe I. Herding dog, their origins and development in Britain. 1st ed. London: Faber and Faber Ltda Press; 1987.

[15] Tafra V, Barrios N, Godoy J, De la Barra R, Gómez M. Primera caracterización morfoestructural y faneróptica del perro ovejero Magallánico, Chile. Arch Zootec. 2014;63: 371–380.

[16] Sociedad Anonima Menendez Behety. Revista Menendez Behety. Feb 1933: 29–32.

[17] Felsenstein J. PHYLIP-Phylogeny Inference Package. Seattle: Department of Genome Sciences, University of Washington; 2005. Available: http://evolution.genetics.washington.edu/phylip.html.

[18] Alexander DH, Novembre J, Lange K. Fast model-based estimation of ancestry in unrelated individuals. Genome Res. 2009;19: 1655–1664. doi: 10.1101/gr.094052.109 19648217 PMC2752134

[19] Pickrell JK, Pritchard JK. Inference of Population Splits and Mixtures from Genome-Wide Allele Frequency Data. PLoS Genet. 2012;8. doi: 10.1371/journal.pgen.1002967 23166502 PMC3499260

[20] Purcell S, Neale B, Todd-Brown K, Thomas L, Ferreira MAR, Bender D, et al. PLINK: A tool set for whole-genome association and population-based linkage analyses. Am J Hum Genet. 2007;81: 559–575. doi: 10.1086/519795 17701901 PMC1950838

[21] Danecek P, Auton A, Abecasis G, Albers CA, Banks E, DePristo MA, et al. The variant call format and VCFtools. Bioinformatics. 2011;27: 2156–2158. doi: 10.1093/bioinformatics/btr330 21653522 PMC3137218

[22] Youatt W. The dog. London: Charles Knight and Co Press; 1845.

[23] Shaw V. The illustrated book of the dog 1879. In: The Sheepdog. British Library Cataloguing-in-Publication Data; 2010. pp. 16–25.

[24] Lee RB. A history and description of the modern dogs of Great Britain and Ireland (non sporting division) including toy, pet, fancy and ladies dogs 1899. In: The Sheepdog. London: British Library Cataloguing-in-Publication Data; 2010. p. 205.

[25] Choudhury A, Hazelhurst S, Meintjes A, Achinike-Oduaran O, Aron S, Gamieldien J, et al. Population-specific common SNPs reflect demographic histories and highlight regions of genomic plasticity with functional relevance. BMC Genomics. 2014;15: 1–20. doi: 10.1186/1471-2164-15-1 24906912 PMC4092225

[26] Hancock AM, Witonsky DB, Alkorta-Aranburu G, Beall CM, Gebremedhin A, Sukernik R, et al. Adaptations to climate-mediated selective pressures in humans. PLoS Genet. 2011;7. doi: 10.1371/journal.pgen.1001375 21533023 PMC3080864

[27] Howat J. Falkland islands to Patagonia. 2015 [cited 19 Oct 2021]. Available: https://patbrit.org/bil/ranchers/jh1.htm.

[28] Imrie J. Recruitment from the Isle of Lewis, Scotland. 2005 [cited 20 Oct 2021]. Available: https://patbrit.org/eng/immig/patrecruit.htm.

[29] Parker HG. Genomic analyses of modern dog breeds. Mamm Genome. 2012;23: 19–27. doi: 10.1007/s00335-011-9387-6 22231497 PMC3559126

[30] Ash E. Dogs: their history and development 1927. In: The Sheepdog. British Library Cataloguing-in-Publication Data; 2010. pp. 59–74.

[31] Briggs R. A history and description of the collie or sheep dog in his British varieties. Horace C, editor. London; 1890.

[32] Luisi P, García A, Berros JM, Motti JMB, Demarchi DA, Alfaro E, et al. Fine-scale genomic analyses of admixed individuals reveal unrecognized genetic ancestry components in Argentina. PLoS One. 2020;15: 1–30. doi: 10.1371/journal.pone.0233808 32673320 PMC7365470

[33] Homburger JR, Moreno-Estrada A, Gignoux CR, Nelson D, Sanchez E, Ortiz-Tello P, et al. Genomic insights into the ancestry and demographic history of south America. PLoS Genet. 2015;11: 1–26. doi: 10.1371/journal.pgen.1005602 26636962 PMC4670080

[34] Flain D. Australian shepherd history: behind the breed. 2020 [cited 19 Oct 2021]. Available: https://www.akc.org/expert-advice/dog-breeds/australian-shepherd-history-behind-breed/.

[35] UK Kennel club. Pastoral Bearded collie. These shaggy coated cattle herders are native to Scotland. 2021 [cited 19 Oct 2021]. Available: https://www.thekennelclub.org.uk/search/breeds-a-to-z/breeds/pastoral/bearded-collie/.

[36] Rigel Border collie. History of Border collie, background. 2020 [cited 19 Oct 2021]. Available: http://www.rigelbordercollies.com/BC_Info.html.

[37] The Cardigan welsh corgi club of America. History of the Cardigan welsh corgi. 2021 [cited 19 Oct 2021]. Available: https://cardigancorgis.com/cwcca/breed/history/.

[38] UK Kennel club. Pastoral Collie (rough). Friendly, happy and active with a glamorous coat and working roots. 2021 [cited 2 Nov 2021]. Available: https://www.thekennelclub.org.uk/search/breeds-a-to-z/breeds/pastoral/collie-rough/.

[39] Australian National Kennel Council. Extended breed standard of the Australian kelpie. 2008 [cited 20 Oct 2021]. Available: https://ankc.org.au/media/pdf/635576344320930744_d0d9014f-a85b-407a-8dc0-d2010a9293e0.pdf.

[40] Hubbard C. Dogs in Britain: a description of all native breeds and most foreign breeds in Britain. 1st ed. London; 1948.

[41] UK Kennel club. Pastoral Shetland sheepdog. Beautifully coated and perfectly sized for an island environment. 2021 [cited 2 Nov 2021]. Available: https://www.thekennelclub.org.uk/search/breeds-a-to-z/breeds/pastoral/shetland-sheepdog/.

[42] Leroy G, Rognon X, Varlet A, Joffrin C, Verrier E. Genetic variability in French dog breeds assessed by pedigree data. J Anim Breed Genet. 2006;123: 1–9. doi: 10.1111/j.1439-0388.2006.00565.x 16420259

[43] Bigi D, Marelli SP, Randi E, Polli M. Genetic characterization of four native Italian shepherd dog breeds and analysis of their relationship to cosmopolitan dog breeds using microsatellite markers. Animal. 2015;9: 1921–1928. doi: 10.1017/S1751731115001561 26245492

[44] Barrios N, Bórquez A, Gómez M, Tafra V, Sponenberg P. Estudio descriptivo del color de manto y señas del perro ovejero Magallánico, Chile. Arch Zootec. 2016;65: 99–101.

[45] Rambaut A. FigTree v.1.4.2, A Graphical Viewer of Phylogenetic Trees. Edinburgh; 2014. Available: http://tree.bio.ed.ac.uksoftwarefigtree.

[46] Browning BL, Browning SR. Improving the accuracy and efficiency of identity-by-descent detection in population data. Genetics. 2013;194: 459–471. doi: 10.1534/genetics.113.150029 23535385 PMC3664855

[47] Team Rs. RStudio: integrated development for R.RStudio. Boston, MA; 2020.

[48] Abraham G, Qiu Y, Inouye M. FlashPCA2: principal component analysis of Biobank-scale genotype datasets. Bioinformatics. 2017;33: 2776–2778. doi: 10.1093/bioinformatics/btx299 28475694

[49] Patterson N, Moorjani P, Luo Y, Mallick S, Rohland N, Zhan Y, et al. Ancient admixture in human history. Genetics. 2012;192: 1065–1093. doi: 10.1534/genetics.112.145037 22960212 PMC3522152

[50] Barbato M, Orozco-terWengel P, Tapio M, Bruford MW. SNeP: A tool to estimate trends in recent effective population size trajectories using genome-wide SNP data. Front Genet. 2015;6: 1–6. doi: 10.3389/fgene.2015.00001 25852748 PMC4367434

[51] Wickham H. ggplot2: Elegant graphics for data analysis. New York; 2016. Available: https://ggplot2.tidyverse.org.

