# Supplementary figures and images for: Patagonian sheepdog: Genomic analyses trace the footprints of extinct UK herding dogs to South America
Source: PLoS Genet. 2022 Apr 28;18(4):e1010160. doi: 10.1371/journal.pgen.1010160 (PMC9049511; doi:10.1371/journal.pgen.1010160)

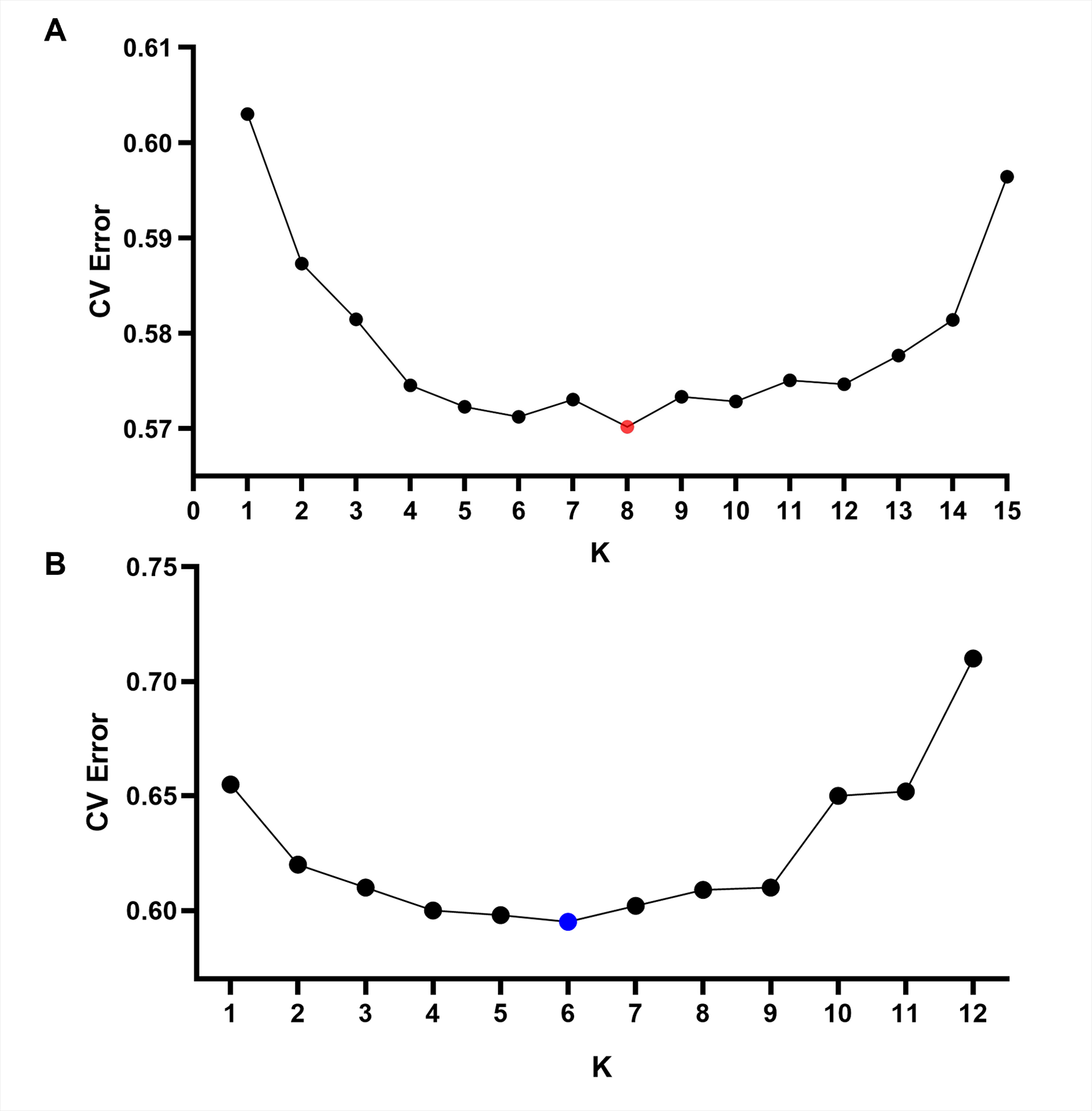

Supplement: S1 Fig — Line graph of CV error values for each ancestry models denoted by K. The upper plot (A) shows the CV error for herding related PGOD dogs, the red dot is the minimal CV error (0.57020). The bottom plot (B) shows the CV error for homogenous PGOD dataset, the blue dot is the minimal CV error (0.59766). (TIF) [file pgen.1010160.s008.tif]

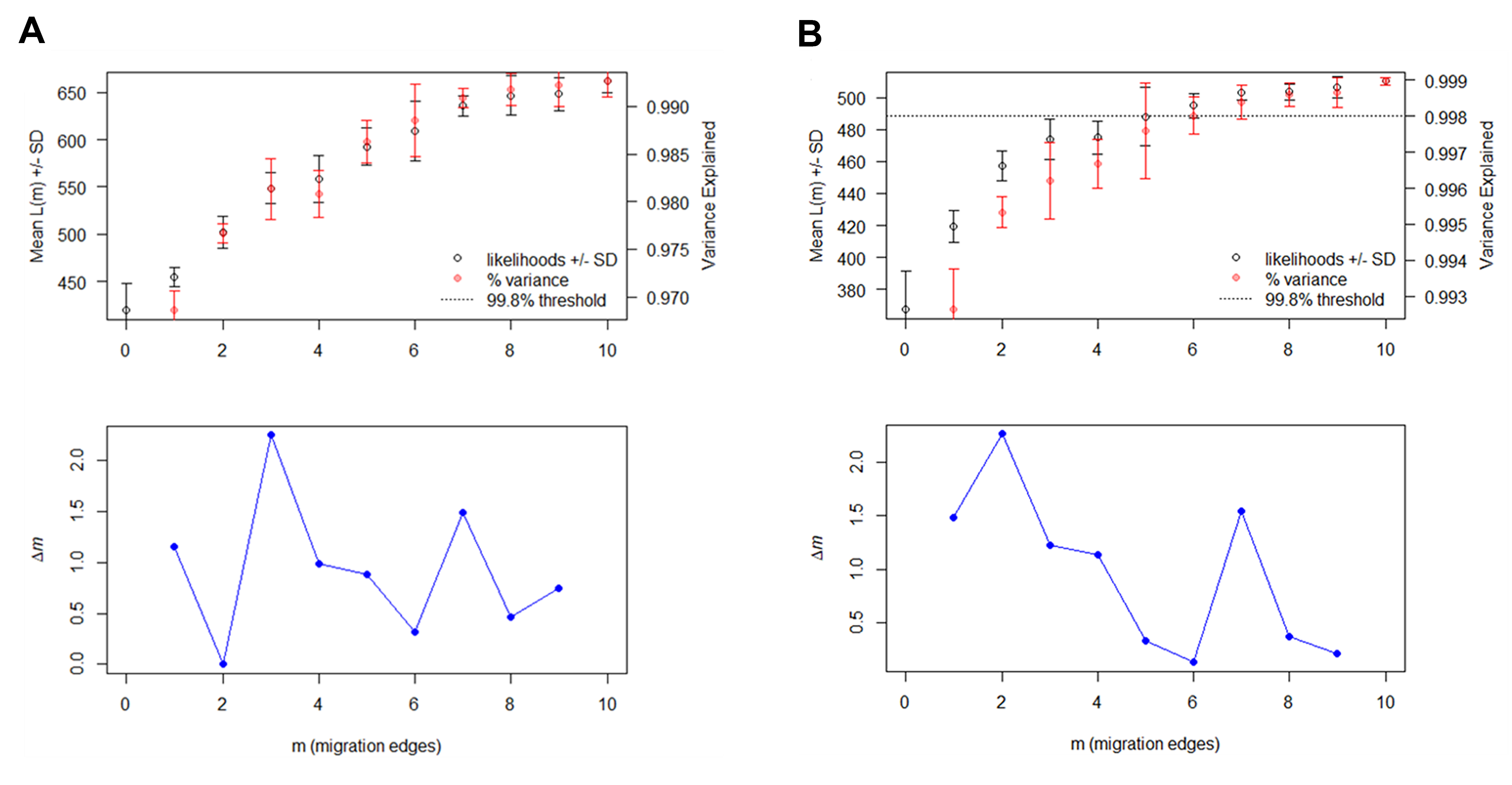

Supplement: S2 Fig — Line plot of the optimal number of migration edges on each population calculated through optM with Treemix output. (A) optM output using Treemix results of subset herding dogs. (B) optM output using Treemix results of subset considering the homogenous PGOD dogs. (TIF) [file pgen.1010160.s009.tif]

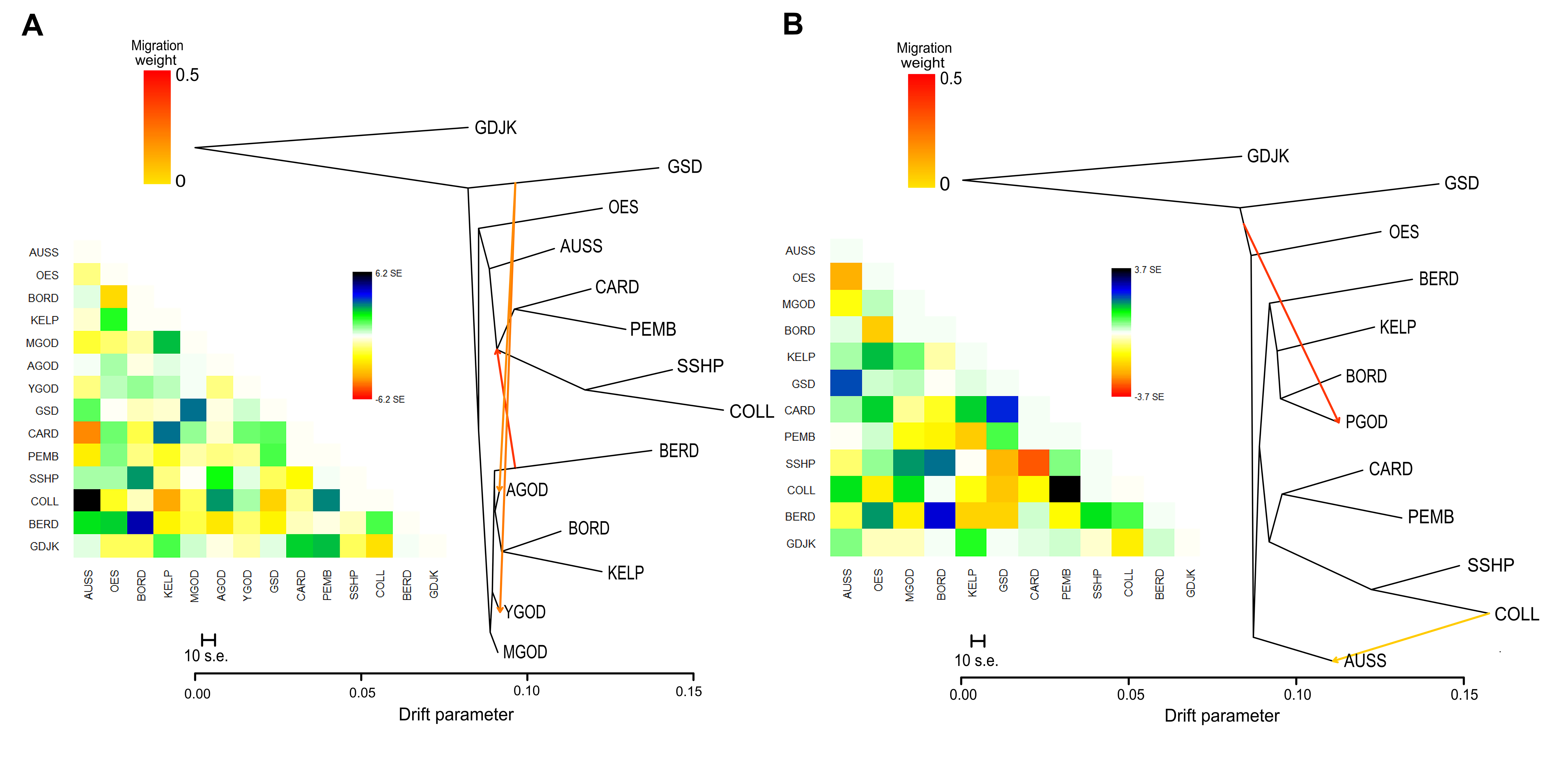

Supplement: S3 Fig — Maximum likelihood trees show the most important migration events. Scale bar shows ten times the average standard error of the sample covariance matrix. The estimated migration between breeds and gene flow are shown according to by direction and weight (yellow to red = 0 to 0.5). (A) Maximum likelihood tree using three migration events within the herding dog subset and PGODs separated by region in AGOD, YGOD, and MGOD. The residual matrix is plotted from a TreeMix analysis under 3 migration events (m = 3). (B) Tree using two migration events within the herding dog subset and the homogenous PGODs. The residual matrix is plotted from a TreeMix analysis under 2 migration events (m = 2). The breed abbreviations correspond to S1 Table. (TIF) [file pgen.1010160.s010.tif]
